# The effectiveness of digital gaming on the functioning and activity of older people living in long-term care facilities: a systematic review and meta-analysis

**DOI:** 10.1007/s40520-023-02459-y

**Published:** 2023-06-20

**Authors:** Saara Kukkohovi, Heidi Siira, Sari Arolaakso, Jouko Miettunen, Satu Elo

**Affiliations:** 1 GeroNursing Centre, Research Unit of Health Science and Technology, University of Oulu, Oulu, Finland; 2 Lapland University of Applied Sciences, Kemi, Finland; 3 Medical Research Center Oulu, Oulu University Hospital and University of Oulu, Oulu, Finland; 4 Oulu University of Applied Sciences, Oulu, Finland

**Keywords:** Digital game, Older people, Functioning, Activity, Long-term care facility

## Abstract

**Background:**

The population is aging globally. Older people living in long-term care facilities have many functional impairments, such as mobility problems and depression. Digital games and so-called exergames can offer a motivating and entertaining way to maintain older people’s physical activity and thus their ability to function. However, previous studies have reported conflicting results about the effects of digital gaming and have focused on community-dwelling older people.

**Objective:**

To identify, critically appraise, and synthesize evidence about the effectiveness of digital games on older people’s physical, psychological, and social functioning and physical and social activity in long-term care facilities.

**Methods:**

Five databases were systematically searched, and relevant studies were screened. Fifteen randomized-controlled trials and quasi-experimental studies (total *N* = 674) were included in meta-analysis.

**Results:**

All digital games used in interventions were exergames. Meta-analysis showed that exergame interventions have a statistically significant large effect on physical functioning [number of studies (*N*) = 6, standardized mean difference (SMD) = 0.97, *p* = 0.001] measured by Timed Up and Go or Short Physical Performance Battery and self-assessed physical activity (*N* = 3, SMD = 1.20, *p* < 0.001) and medium effect on social functioning (*N* = 5, SMD = 0.74, *p* = 0.016) compared to alternative intervention or no intervention. Social activity was not measured in any study.

**Conclusions:**

The results are encouraging that exergames effectively increase the functioning and activity of older adults living in long-term facilities. Successful implementation of such activities requires the competence of nursing staff and rehabilitation professionals in digitalization.

## Introduction

The population is aging globally, and the number of older people is estimated to more than double from 2020 to 2050, when 16% of the world’s population will be over 65 years old [1]. As the number of older people is growing, the number of people living in long-term care facilities is also increasing. Older people living in long-term care facilities are more likely to be functionally and cognitively impaired than their independent peers [2]. Living in long-term care facilities has been found to cause a decline in physical functioning [3], and it has also been found to result in experiencing symptoms of depression, loneliness, and social isolation in older people [2, 4].

The physical activity of older people is related to physical and psychological functioning and functional impairments [5, 6]. Exercise and even light physical activity have a positive effect on the physical functioning of older people and coping with activities of daily living (ADL) for older people living in assisted living facilities [7–9]. Physical activity has also been found to smooth out the worsening of depressive symptoms in care home residents [6]. Higher physical activity is also correlated with a higher quality of life of older people [10]. However, residents in long-term care environments have many barriers to physical activity, which can affect their motivation to exercise [11]. The fun, enjoyment, and sociability of training are big motivators for exercise among older people [12, 13], and digital games and exergaming enable these dimensions [14].

Digital games are games played on a digital device. A digital device can be, for example, a computer, a game console, a tablet computer, or a smartphone. According to previous reviews studying digital games, Nintendo Wii and Microsoft Kinect have been the most used gaming systems by older people [15]. Playing these kinds of active video games where the player uses one’s own body movement is often called exergaming [14]. Playing digital games that train balance, coordination, physical performance, and physical activity has been found to have a positive effect on the physical and psychological functioning of older people by reducing the experience of depression and improving balance. According to the previous studies, older people find digital games to be more motivating and entertaining than normal physical activities [16, 17]. Playing exergames is often social experience and it offers an opportunity for playfulness and social interaction [18]. This increased interaction with others during the play can decrease loneliness and strengthen social connection also among older people [18, 19]. Social isolation has been found to increase cognitive decline [20] and low cognitive skills hinder, among other things, older peoples’ coping with daily activities. There are already several systematic reviews about effects of exergaming on cognition of older adults that show the benefits of exergames having a positive influence on processing speed, working memory, and executive function [21].

Previous studies have also obtained conflicting results regarding the effects of digital gaming [22, 23]. The existing literature and earlier systematic reviews of digital gaming have focused on the home environment, and digital games have not been studied much in the long-term care environment [24]. More research is therefore needed on the effects of playing digital games on the functioning and activity of older people in long-term care.

The objective of this review was to identify, critically appraise, and synthesize evidence about the effectiveness of playing digital games on older people’s physical, psychological, and social functioning and physical and social activity in long-term care facilities. Two research questions were addressed:

1. What digital gaming interventions have been conducted for older people in long-term care facilities?
2. What is the effect of digital gaming versus usual care or conventional exercise on older people’s functioning and activity in long-term care facilities?

## Methods

A systematic review and meta-analysis were conducted according to Joanna Briggs Institute guidelines [25]. The Preferred Reporting Items for Systematic Reviews and Meta-analysis (PRISMA) statement was applied for reporting the review [26]. The review was registered in PROSPERO (CRD42022307491). No protocol was published for this systematic review.

### Search strategy

Data retrieval was carried out by an informant scientist. PubMed, Web of Science, CINAHL (EBSCO), Scopus, and Cochrane electronic databases were used to retrieve studies published up to November 2021. No publication period restrictions were made. Key Medical Subject Headings (MESH) terms and CINAHL headings were applied in the searches whenever possible. Search terms for research question one combined the following subject headings and keywords, formatted according to the requirements for each database: digital game, older people, and long-term care. Search terms for research question two combined the following subject headings and keywords, formatted according to the requirements for each database: digital game, older people, functioning, social activity, and physical activity. Synonyms were combined using the OR operator, and different search terms were combined using AND operator. NOT operator was used to exclude children and adolescents. The complete search terms for each database are presented in Appendix Table 4. A manual search was conducted in the reference lists of the articles included. No new references were found by manual searches.

### Study selection and data extraction

Two reviewers (SK and either SE, SA, or HS) independently screened records for inclusion in the title and abstract phase and in the full-text phase. Selection was made based on the inclusion and exclusion criteria created according to the PICO format. Full-text original research articles were eligible. Study inclusion and exclusion criteria were as follows: older people without specific diseases excluding memory disorders living in long-term care (population), digital gaming interventions (intervention), control group with usual care or alternative intervention (comparator), and change in measured physical, psychological, or social functioning and/or change in physical or social activity from baseline to the last available follow-up (outcome). Studies measuring physical functioning using the Timed Up and Go (TUG) test or Short Physical Performance Battery (SPPB) were chosen for this review. These tests were chosen, because they measure a wide area of physical functioning, considering balance, strength, agility, and walking speed. The measures used for mental and social functioning and activity were not limited. Interventions that combined digital gaming with, for example, conventional exercise or physical therapy were not eligible. Only studies published in English were included due to the lack of resources for the translation of other languages. Reviewers were blinded to each other’s decisions and conflicts were solved in the consensus of two reviewers. If the consensus was not reached, a third reviewer was consulted. Data extraction was done by one researcher (SK), and another researcher (SE) checked the quantitative data. Data selection and extraction were done and recorded via Covidence systematic review software.

### Quality assessment

A quality assessment was made for 15 studies that met the inclusion criteria in accordance with the quality assessment criteria of the Joanna Briggs Institute. Quality assessment was made by two independent reviewers (SK and either SE, SA, or HS). Disagreements were solved in the conclusion of two reviewers. Studies were accepted for the review if 50% of the assessment criteria were met. All 15 studies were accepted for the review. The quality assessment for RCT studies is presented in Table 1 and for quasi-experimental studies in Table 2.

**Table 1:** Quality assessment scores of the selected studies according to Joanna Briggs RCT studies

Authors	1	2	3	4	5	6	7	8	9	10	11	12	13	Total
Babadi Daneshmandi [37]	Y	?	Y	N/A	N/A	?	Y	Y	N/A	Y	?	Y	Y	7/11
Delbroek et al. [28]	Y	?	Y	N/A	N/A	Y	Y	Y	N	Y	Y	Y	Y	9/11
Fakhro et al. [34]	Y	?	?	N/A	N/A	Y	Y	N	N	Y	Y	Y	Y	7/11
Mugueta-Aguinaga & Garcia-Zapirain [31]	Y	?	Y	N/A	N/A	?	Y	Y	N	Y	?	Y	Y	7/11
Stanmore et al. [33]	Y	?	Y	N/A	N/A	N	Y	Y	Y	Y	Y	Y	Y	9/11
Swinnen et al. [29]	Y	?	Y	N/A	N/A	Y	Y	Y	N	Y	Y	Y	Y	9/11

Rating scale: Yes (Y), No (N), Unclear (?), Not applicable (NA)

1. Was true randomization used for the assignment of participants to treatment groups?

2. Was allocation to treatment groups concealed?

3. Were the treatment groups similar at the baseline?

4. Were participants blind to the treatment assignment?

5. Were those delivering treatment blind to the treatment assignment?

6. Were outcome assessors blind to the treatment assignment?

7. Were the treatment groups treated identically other than the intervention of interest?

8. Was follow-up completed, and if not, were differences between groups in terms of their follow-up adequately described and analyzed?

9. Were participants analyzed in the groups to which they were randomized?

10. Were outcomes measured in the same way for the treatment groups?

11. Were outcomes measured in a reliable way?

12. Was an appropriate statistical analysis used?

13. Was the trial design appropriate, and were any deviations from the standard RCT design (individual randomization, parallel groups) accounted for in the conduct and analysis of the trial?

**Table 2:** Quality assessment scores of the selected studies according to Joanna Briggs quasi-experimental studies

Authors	1	2	3	4	5	6	7	8	9	Total
Chen et al. [35]	Y	N	?	Y	Y	Y	Y	?	Y	6/9
Cicek et al. [32]	Y	Y	?	Y	Y	N	Y	Y	Y	7/9
Janssen et al. [30]	Y	Y	Y	Y	Y	N	Y	Y	Y	8/9
Jung et al. [39]	Y	?	Y	Y	Y	Y	Y	Y	Y	8/9
Keogh et al. [42]	Y	?	?	Y	Y	N	Y	Y	Y	6/9
Padala et al. [38]	Y	Y	Y	Y	Y	Y	Y	Y	Y	9/9
Ramnath et al. [41]	Y	N	?	Y	Y	Y	Y	Y	Y	7/9
Rica et al. [40]	Y	?	Y	Y	Y	?	Y	?	Y	6/9
Soares et al. [39]	Y	Y	?	Y	Y	N	Y	?	Y	6/9

Rating scale: Yes (Y), No (N), Unclear (?), Not applicable (NA)

1. Is it clear in the study what is the ‘cause’ and what is the ‘effect’ (i.e., there is no confusion about which variable comes first)?

2. Were the participants included in any comparisons similar?

3. Were the participants included in any comparisons receiving similar treatment/care, other than the exposure or intervention of interest?

4. Was there a control group?

5. Were there multiple measurements of the outcome both pre- and post-intervention/exposure?

6. Was follow-up complete, and if not, were differences between groups in terms of their follow-up adequately described and analyzed?

7. Were the outcomes of the participants included in any comparisons measured in the same way?

8. Were outcomes measured in a reliable way?

9. Was the appropriate statistical analysis used?

### Data analysis and synthesis

Narrative synthesis and meta-analysis were conducted for all 15 studies. Separate meta-analyses were performed for RCT studies and quasi-experimental studies and for different outcomes. The level of statistical significance was *p* < 0.05. Post-intervention means and SD values of continuous outcomes were used to calculate standardized mean differences (SMD) and 95% confidence intervals (CIs) between groups with Cohen’s d value. If the study had multiple experimental or comparator groups, the best matching group was chosen for meta-analysis, usually the passive control group. Mean, standard deviations, and sample sizes were estimated when they were not directly reported. A random-effects model was used for the meta-analysis due to differences between interventions and populations. The effect size was considered small (SMD = 0.2 to 0.5), medium (SMD = 0.5 to 0.8), or large (SMD > 0.8). Statistical heterogeneity was observed with the Chi^2^ test and *I*^2^ test. The significance level of the Chi^2^ test was *p* < 0.10, indicating heterogeneity. In the *I*^2^ test, the value of 0–40% would be considered low heterogeneity, 30–60% indicates moderate heterogeneity, 50–90% indicates substantial heterogeneity, and 75–100% indicates considerable heterogeneity [27]. Publication bias could not be assessed with statistical methods due to the small number of studies per outcome.

## Results

### Search outcomes

**Fig. 1:**
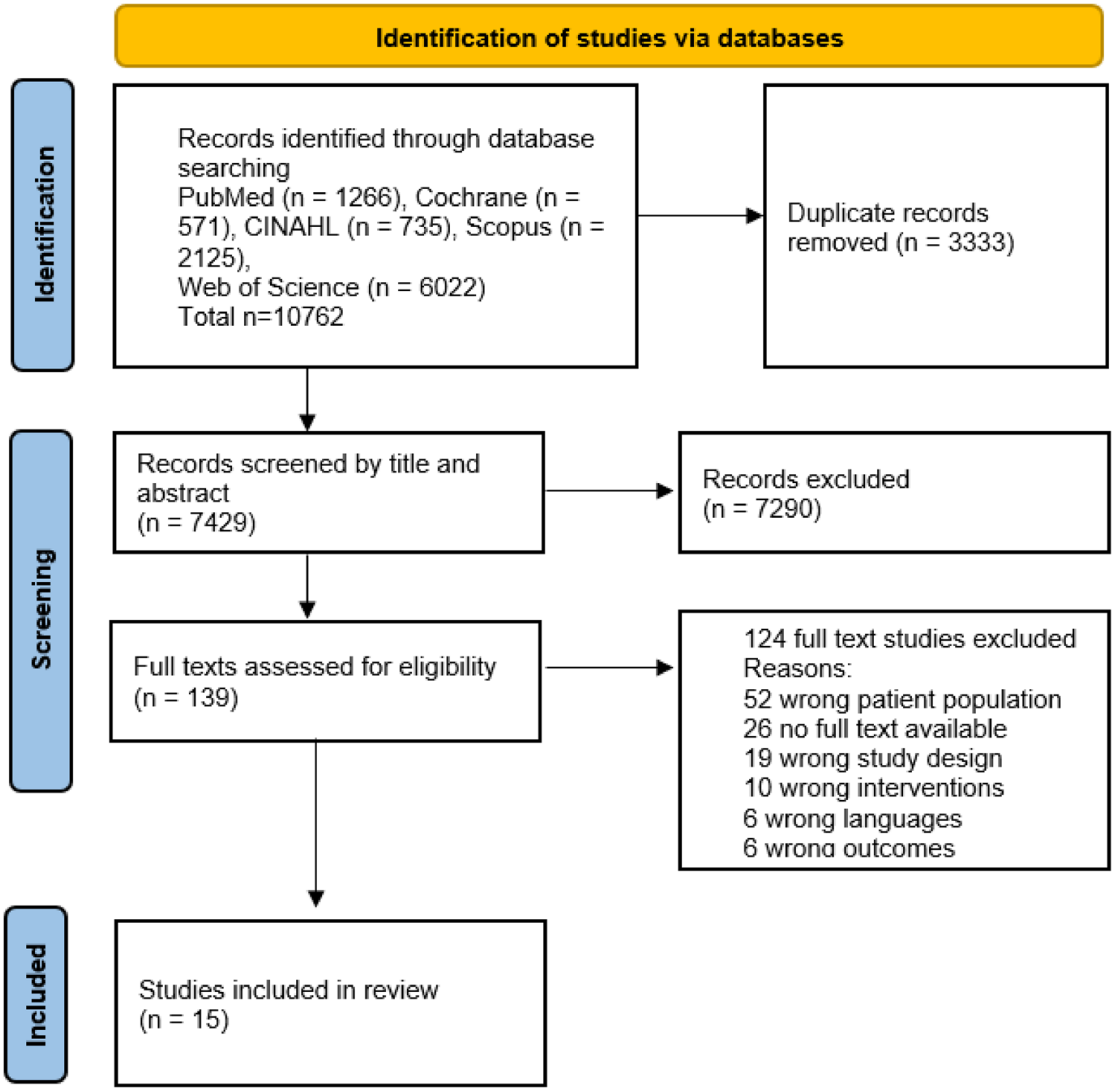
PRISMA flow diagram of the article selection process

The search yielded 10,762 references, and after removing duplicates, 7429 references remained. Then, 139 full texts were assessed. A total of 124 studies were excluded, and 15 articles were included for the review. The search outcomes are shown in Fig. 1.

### Study characteristics

Studies selected for this review were conducted between 2009 and 2021 in Belgium [28, 29], The Netherlands [30], Spain [31], Turkey [32], The United Kingdom [33], Lebanon [34], Taiwan [35], Singapore [36], Iran [37], USA [38], Brazil [39, 40], South Africa [41], and Australia [42]. Six studies were RCTs [28, 29, 31, 33, 34, 37], and nine were quasi-experimental studies [30, 32, 35, 36, 38–42]. The sample sizes ranged from 19 to 106 participants (*N* = 674). The mean age of the study participants ranged from 66.5 to 87.5 years. In four studies, participants had cognitive disorders like memory complaints or Alzheimer’s disease [28, 29, 38, 41]. In one study, participants had poor balance [37]; in two studies, participants had frailty syndrome or risk for frailty syndrome [29, 39]; and in one study, all participants were women [40]. A summary of the participants, measures, intervention(s), control, and results is given in Table 3.

**Table 3:** Study characteristics

Authors/year/country	Study design	Participants	Measures	Intervention(s)	Control	Results
Babadi and Daneshmandi 2021 Iran [37]	RCT	EG1 (*n* = 12) mean age 66.5 EG2 (*n* = 12) mean age 67.5 CG (*n* = 12) mean age 66.75	TUG	EG1: Xbox Kinect sports EG2: Progressive conventional balance training 3 × 1 h/week for 9 weeks	Usual care	TUG: EG1: Improved EG2: Improved CG: No change EG1/EG2: *p* = 1.000 EG1/CG: *p* = 0.014 EG2/CG: *p* = 0.038
Cicek et al. 2020 Turkey [32]	Quasi-experimental study	EG1 (*n* = 16) mean age 72.3 EG2 (*n* = 14) mean age 75.1 CG (*n* = 14) mean age 73.9	TUG HRSD WHOQLQ	EG1: Nintendo Wii Fit Plus EG2: Individual training program (10 min of cycling and 20 min of walking on a treadmill) 2 × 30 min/week for 8 weeks	Usual care	TUG: EG1: Improved EG2: No change CG: No change EG1/EG2: *p* = 0.007 EG1/CG: *p* = 0.001 EG2/CG: *p* = 0.578 HRSD: EG1: Improved EG2: Improved CG: No change *p* = 0.038 WHOQOL-BREF EG1: No change EG2: No change CG: No change *p* = 0.939
Delbroek et al. 2017 Belgium [28]	RCT	EG (*n* = 8) mean age 86.9 CG (*n* = 9) mean age 87.5	iTUG	BioRescue + usual care 2 × 18–30/week for 6 weeks	Usual care	iTUG: EG: Improved CG: No change
Fakhro et al. 2020 Lebanon [34]	RCT	Overall mean age 72.2 years EG (*n* = 30) CG (*n* = 30)	TUG	Nintendo Wii fit 3 × 40 min/week for 8 weeks	Usual care	TUG: EG: Improved CG: Got worse EG/CG: *p* < 0.001
Janssen et al. 2013 Netherlands [30]	Quasi-experimental study	EG1 (*n* = 8) median age 84.5 EG2 (*n* = 8) median age 81.5 CG (*n* = 13) median age 80.0	LAPAQ	EG1: Experienced Nintendo Wii Fit Plus players EG2: Novice Nintendo Wii Fit Plus players 2 × 1 h (10–15 min per participant)/week for 12 weeks	Usual care	LAPAQ: EG1: Improved EG2: Improved CG: Improved EG1/EG2: Not significant EG1/CG: *p* = 0.014 EG2/CG: *p* = 0.005
Jung et al. 2009 Singapore [36]	Quasi-experimental study	EG (*n* = 30) CG (*n* = 15)	UCLA Loneliness Scale PAQ	EG1: Nintendo Wii sports and cooking mama EG2: traditional games such as memory games, Jenga, and UNO 3× 1.5 h (15 min per participant)/week for 6 weeks	–	UCLA: EG1: Improved EG2: Got worse *p* < 0.01 PAQ: EG1: Improved EG2: Got worse *p* < 0.01
Keogh et al. 2014 Australia [42]	Quasi-experimental study	EG (*n* = 13) mean age 81 CG (*n* = 13) mean age 85	WHOQOL-BREF RAPA	Nintendo Wii Sports Average playtime 30 min/week for 8 weeks	Usual care	WHOQOL-BREF: EG: No change CG: No change *p* = 0.483 RAPA: EG: Improved CG: Got worse *p* = 0.009
Mugueta-Aguinaga & Garcia-Zapirain 2017 Spain [31]	RCT	EG (*n* = 20) mean age 85 CG (*n* = 19) mean age 83	SPPB	FRED game 3 × 20 min/week for 3 weeks	Usual care	SPPB: EG: Improved CG: Got worse
Padala et al. 2012 USA [38]	Quasi-experimental study	EG (*n* = 11) mean age 79.3 CG (*n* = 11) mean age 81.6	TUG	EG1: Nintendo Wii Fit EG2: Walking exercise (indoors) 5 × 30 min/week for 8 weeks	–	TUG: EG1: No change EG2: No change *p* = 0.52
Ramnath et al. 2021 South Africa [41]	Quasi-experimental study	EG (*n* = 23) mean age 70.8 CG (*n* = 22) mean age 74.14	TUG	EG1: Xbox-360 Kinect Sports 2 × 1 h (30 min playtime)/week for 12 weeks EG2: Low-intensity conventional multimodal exercise 2 × 1 h/week for 12 weeks	–	TUG: EG1: Improved EG2: No change *p* < 0.001
Rica et al. 2020 Brazil [40]	Quasi-experimental study	Aged women (*n* = 50) over 60 years old	BDI WHOQOL-BREF	EG1: Xbox-360 Kinect Sports EG2: Board games + normal daily activities 3 × 1 h/week for 12 weeks	–	BDI: EG1: Improved EG2: Got worse *p* < 0.001 WHOQOL-BREF: EG1: Improved EG2: No change *p* < 0.05
Soares et al. 2016 Brazil [39]	Quasi-experimental study	EG (*n* = 11) mean age 83 CG (*n* = 8) mean age 80	TUG	SIRTET 2× 15–25 min/week for 12 weeks	Usual care	TUG: EG: Improved CG: No change
Stanmore et al. 2019 UK [33]	RCT	EG (*n* = 56) mean age 77.9 CG (*n* = 50) mean age 77.8	TUG GDS PASE	Exergame (MIRA-system) + standard care 3 × /week for 12 weeks	Usual care (community fall prevention and exercise advice by physiotherapists)	TUG: EG: No change CG: No change GDS: EG: No change CG: No change *p* = 0.34 PASE: EG: No change CG: No change *p* = 0.91
Swinnen et al. 2021 Belgium [29]	RCT	EG (*n* = 23) mean age 84.7 CG (*n* = 22) mean age 85.3	SPPB CSDD	EG1: Dividat Senso + Usual care (pharmacotherapy and physiotherapy) EG2: Watching and listening to favorite music videos + Usual care (pharmacotherapy and physiotherapy) 3 × 15 min/week for 8 weeks	–	SPPB: EG: Improved CG: Got worse *p* < 0.001 CSDD: EG1: Improved EG2: Got worse *p* < 0.001
Chen et al. 2012 Taiwan [35]	Quasi-experimental study	EG (*n* = 22) mean age 78.55 CG (*n* = 39) mean age 79.52	quasi-HRQOL questionnaire	Xbox-360 Kinect 3 × 30 min/week for 4 weeks	Usual care	Quasi-HRQOL: EG: Improved CG: No change *p* < 0.01

*RCT * randomized-controlled trial, *EG* experimental group, *CG* control group.

Abbreviations of measures are explained in section “Outcome measures”

### Risk of bias

The quality assessment points of the RCT studies ranged from 7 to 9 points (Table 1). Randomization was used in all six studies, but the concealment of allocation to treatment groups was unclear in all six studies. Blinding of participants and personnel was not possible due to the nature of the interventions. Outcome assessors were blinded in three studies [28, 29, 34]. The assessment point average was 8/11, so all RCT studies were of high methodological quality.

In quasi-experimental studies, assessment points ranged from 6 to 9 points (Table 2). The assessment point average was 7/9, indicating high methodological quality. In two studies, participants were not similar in the two groups due to gender [35] or function for activities of daily living [40]. In three studies, this assessment criteria was unclear, because participants’ characteristics were not presented clearly [36, 40, 42]. In five studies, follow-up was not complete and reasons for dropouts were unclear or the effect of dropouts on the results was not analyzed [30, 32, 39, 40, 42]. Randomization of participants was not done in quasi-experimental studies, and in three trials, participants chose the group they wanted to join [32, 35, 39].

### Characteristics of the described interventions

All digital games used in the interventions were exergames. In six studies, the gaming console was Nintendo Wii [30, 32, 34, 36, 38, 42]. The Microsoft Xbox-360 Kinect console was used in four studies [35, 37, 40, 41]. In three studies, participants played exergames that utilized Kinect motion sensors. The games were FRED [31], SIRTET [39], and MIRA-system [33]. In two studies, games utilized a force platform to control games [28, 29]. These games were designed to improve, for example, balance, strength, and flexibility.

In three studies, participants were divided into three groups [30, 32, 37]. One study compared exergaming to conventional balance training and usual care [37], one to conventional exercise and usual care [32], and one compared experienced Wii players to inexperienced players and to usual care [30]. Twelve studies divided participants into two groups. In six studies, exergaming interventions were compared to usual care [28, 31, 34, 35, 39, 42]. Two studies compared exergaming to playing traditional games [36, 40]. Padala et al. [38] and Ramnath et al. [41] compared exergaming to conventional exercise. In one study, controls listened to favorite music [29], and in one study, they received standard community fall prevention advice and an exercise leaflet [33].

Interventions lasted from 3 to 12 weeks, with 8 and 12 weeks being the most popular ones. Participants played games from two to five times per week, and in most of the studies (8), participants played games three times per week. Gaming sessions lasted from 15 min to 1.5 h. In one study, participants selected the frequency, duration, and type of games [42]. In six interventions, participants played games individually [29, 31, 32, 38–40], in one intervention, games were played with a partner in a group of two pairs [41], in three interventions in group [30, 36, 42], and in one study individually or in a group [33]. Four studies did not report whether the games were played in a group or individually [28, 34, 35, 37].

### Outcome measures

Physical functioning was measured by the TUG test and SPPB. Psychological functioning was measured by the Hamilton Rating Scale for Depression (HRSD), Beck Depression Inventory (BDI), Geriatric Depression Scale (GDS), and Cornell Scale for Depression in Dementia (CSDD). Social functioning was measured by the Word Health Organization Quality of Life short-form (WHOQOL-BREF) questionnaire and the quasi-HRQOL questionnaire. Only the social functioning domains of the quality-of-life questionnaire were included in this review. Loneliness was measured by the UCLA loneliness scale. Physical activity was measured by the Physical Activity Scale for the Elderly (PASE), Rapid Assessment of Physical Activity (RAPA), Physical Activity Questionnaire for Elderly Japanese, and the LASA Physical Activity Questionnaire (LAPAQ).

### Effectiveness of interventions

Ten studies assessed changes in physical functioning measured by TUG [28, 32–34, 37–39, 41] or SPPB [29, 31]. Eight of the ten studies reported significant positive changes in TUG or SPPB scores in the exergame group [28, 29, 31, 32, 34, 37, 39, 41]. Five studies reported significant between-group changes in favor of exergaming compared to alternative intervention or control groups [29, 32, 34, 37, 41]. In three studies, physical functioning in the exergaming group improved and the control group did not, but those studies did not report between-group *p *values [28, 31, 39]. Two studies did not find statistically significant inter-group or between-group differences [33, 38].

Psychological functioning was measured in four studies using different depression scales [29, 32, 33, 40]. Three of the four studies reported a positive change in depression scale scores of the exergame group after intervention [29, 32, 40], while the alternative intervention group or control group did not improve. In Cicek et al. [32], the conventional exercise group scores improved, but the control group did not. Two studies reported significant between-group changes in favor of exergaming [29, 40].

Social functioning was measured in five studies [32, 35, 36, 40, 42]. In three studies, results in exergaming groups improved, but control/alternative intervention groups did not improve [35, 36, 40]. In Cicek et al. [32], exergaming group, conventional exercise group, and control group results improved, but the improvement was not statistically significant. Between-group comparison revealed that three studies had statistically significant differences between the experimental group and the alternative intervention group or control group in favor of exergaming [35, 36, 40].

Four studies measured changes in physical activity [30, 33, 36, 42]. All four studies reported increased levels of physical activity in exergaming groups, but in Stanmore et al. [33], the results were not statistically significant. In three studies, the exergame group improved more than the alternative intervention or control group in between-group comparison [30, 36, 42].

#### Meta-analysis

A meta-analysis was conducted on six RCT studies (number of individuals, *n* = 278) that investigated the effect of exergaming on physical functioning [28, 29, 31, 33, 34, 37]. A meta-analysis on pooled TUG and SPPB scores showed a large effect in favor of exergaming (SMD = 0.97, 95% CI [0.42, 1.52], *p* = 0.001). A meta-analysis was conducted on four quasi-experimental studies (*n* = 116) that investigated the effects of exergaming on physical functioning as measured by TUG [33, 38, 39, 41]. A meta-analysis of pooled TUG scores showed that there is no significant difference between exergaming and comparator groups (SMD = 0.34, 95% CI [0.17, 0.86], *p* = 0.19).

A meta-analysis was conducted on two RCT studies (*n* = 137) that investigated the effects of exergaming on depression [29, 33]. A meta-analysis of pooled depression scale scores showed that there is no significant difference between groups (SMD = 0.52, 95% CI [− 0.59, 1.63], *p* = 0.35). Another meta-analysis was conducted on two quasi-experimental studies (*n* = 40) that investigated the effects of exergaming on psychological functioning (depressive symptoms) [32, 40]. A meta-analysis of pooled depression scale scores showed that there is no significant difference between groups (SMD = 1.46, 95% CI [− 0.46, 3.38], *p* = 0.14).

A meta-analysis was conducted on five quasi-experimental studies (*n* = 192) that investigated the effects of exergaming on social functioning [32, 35, 36, 40, 42]. A meta-analysis of pooled quality-of-life social domain scores and loneliness scale scores showed a medium effect in favor of the exergaming group (SMD = 0.74, 95% CI [0.14, 1.35], *p* = 0.016).

**Fig. 2:**
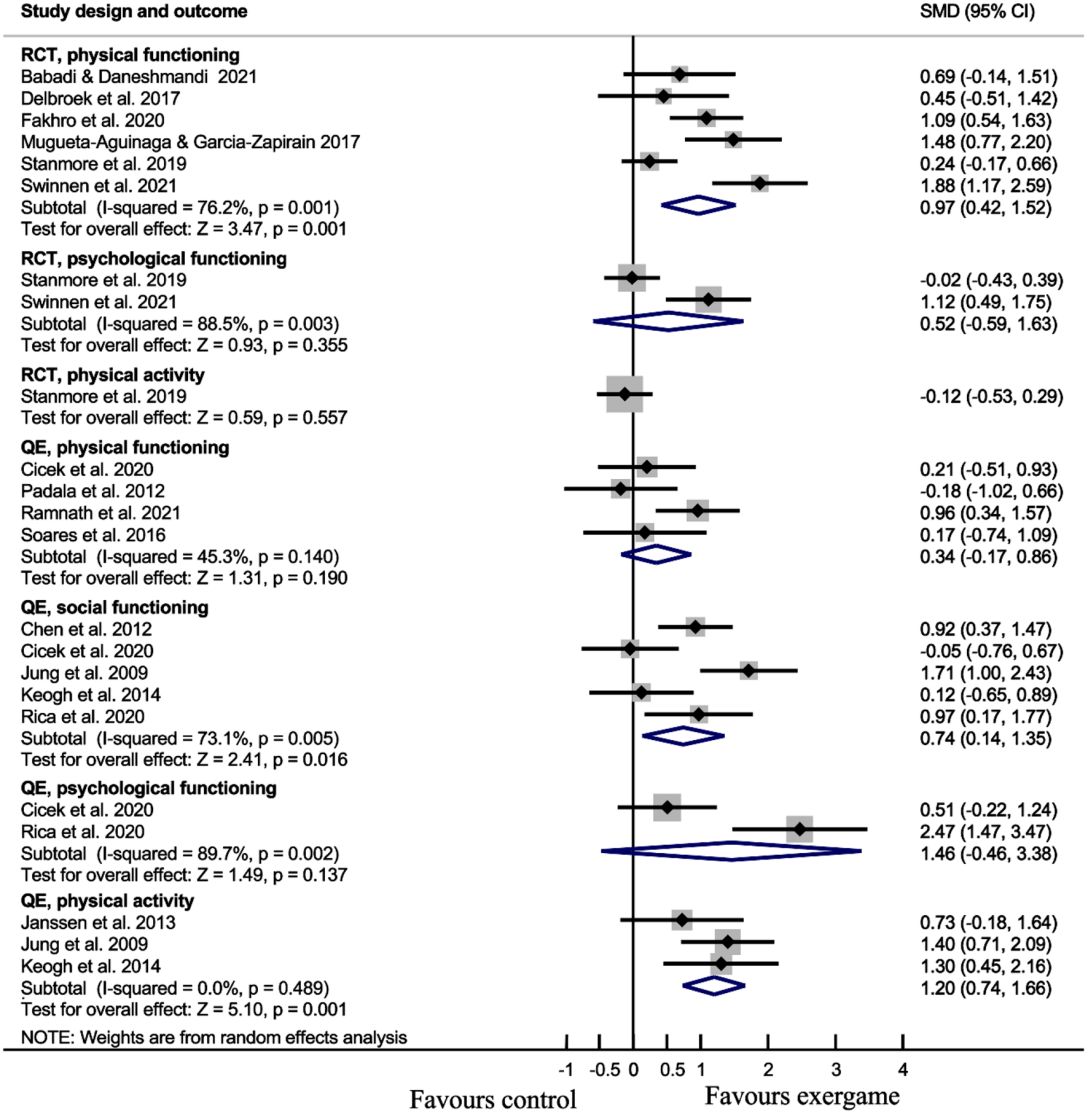
Forest plot of pooled effect sizes of individual studies

A meta-analysis was conducted on three quasi-experimental studies (*n* = 91) that investigated the effects of exergaming on physical activity [30, 38, 42]. A meta-analysis of pooled physical activity scale scores showed a large effect in favor of exergaming (SMD = 1.20, 95% CI [0.74, 1.66], *p* < 0.001). The results of the meta-analysis are shown in Fig. 2.

## Discussion

Based on the results of this review, exergaming has a positive effect on the physical functioning of long-term care residents measured by TUG or SPPB. In previous reviews, it has also been found that exergaming has a positive effect on the balance, mobility, and walking of older people [43–45]. Unlike in this review, those studies also included older people living at home and people who used gaming, for example, for rehabilitation of Parkinson’s disease. In a meta-analysis performed by Taylor et al. [22], there were no statistically significant differences in TUG scores when exergames were compared with no intervention or with conventional exercise. Based on the results of this review, exergaming seems to be effective in improving walking speed, lower limb muscle strength, and balance in older people with memory disorders [28, 29, 41], which supports the results of a previous study [46]. In studies where participants had already diagnosed frailty syndrome or poor balance, TUG scores improved after exergaming intervention. This finding is in line with the previous research [47].

Earlier research has reported mixed results regarding the effects of exergaming on older adults’ mood and depression. In Zeng et al. [47], exergaming did not affect the mood of older people, but in Yen & Chiu [48], exergaming had a large effect on older adults’ depression. In this review, results were also mixed. In three of four studies, depression scale scores were statistically significantly lower after exergaming intervention, indicating fewer symptoms of depression [29, 32, 40]. However, the meta-analysis of RCT studies or quasi-experimental studies comparing exergaming to alternative intervention or no intervention did not reach the limit of a statistically significant difference. Previously, exercising has been found to reduce the symptoms of depression in older people [49, 50]. Also, in this study, four out of five experimental groups doing physical exercises or playing exergames improved in depression scores, but among participants in physically passive intervention or control groups, the scores did not improve. Only four studies measured the effects of exergaming on depression, so this field needs more empirical research.

Social isolation has many adverse health effects, including a higher risk of mortality [51], so a variety of interventions have been implemented for older people to reduce loneliness and increase social interaction [52]. Playing exergames with a partner has been found to reduce loneliness of the older people [53]. In this current review, participants’ loneliness was measured only in one study, and in the other four studies, social functioning was measured as a part of the quality-of-life measurement. Vázquez et al. [54] also found that none of the studies included in their review focused on social health. However, their meta-analysis showed that participants in the exergame group experienced higher beneficial effects from video game-based interventions than those in the control group. In addition, Li et al. [19] summarized that exergames could be an effective intervention for social improvements among older adults and gaming has been also found to reduce social anxiety and increase sociability [55]. A meta-analysis conducted in this review revealed a medium effect of exergaming intervention on the social functioning of older people. Exergaming interventions improved social relationships and reduced loneliness in three studies [35, 36, 40], while playing traditional games and board games did not [36, 40]. More research is needed about the effects of exergaming on social functioning measured by different scales, the ones focusing more on social participation, for example, the Social Engagement Scale. In the future, whether playing in a group is more effective in terms of social functioning than playing alone should also be investigated.

Kahlbaugh et al. [53] found that older people with a positive mood are more physically active and feeling less lonely is the greatest predictor of a positive mood. In the current review, playing Nintendo Wii Fit and Sports games increased the physical activity of older people living in long-term care. In all three studies where physical activity increased, exergames were played in groups [30, 36, 42]. In Carrasco et al. [56], playing Nintendo Wii Sports did not increase the physical activity of community-dwelling older people. Independently living older people are probably more active in their everyday life than long-term care residents, so exergaming intervention might be more effective on this less active population. Longitudinal research about the maintenance of physical activity after the intervention period would be important to study in the future. In all studies selected for this review, physical activity was measured by a self-reported questionnaire. None of the studies used an activity bracelet that would measure activity objectively.

None of the studies included in this review compared outcome results between gender. Therefore, there is no evidence on whether exergaming is more beneficial for other gender. In 9 of the 15 studies, majority of the participants were women, but there were no gender differences in the groups compared in the studies. Because women live longer than men, most long-term care facility residents are usually women, which explains the uneven gender distribution in the studies.

### Strengths and limitations

The strength of this review is that it synthesizes the effects of digital gaming on the wide-ranging functional ability of older people. We did not limit the interventions to a specific game or game device. There are some limitations to this study. Interventions, comparator groups, and participants varied widely across the studies, so the real effect is challenging to assess. A random-effect model was used in the meta-analysis to correct these effects. Studies selected for this review had small sample sizes, and only three had 60 or over 60 participants, which might lower the statistical power of the studies. We did not restrict studies to randomized-controlled trials, and we also included quasi-experimental studies. This might have caused bias in this study. However, RCT studies and quasi-experimental studies were separated in the meta-analysis. Only a few of the included studies examined psychological and social functioning, and social functioning was mainly measured as part of the quality of life. In two meta-analyses of psychological functioning, there were only two studies in both analyses. Therefore, social and psychological functioning results should be interpreted with caution. We included studies published only in English; therefore, studies that would have met the inclusion criteria may have been left out of this review.

## Conclusions

Digital gaming interventions implemented in the long-term care context for older people focus on exergaming, and Nintendo Wii and XBox 360 Kinect are the most common game devices. Playing digital exergames seems to be effective in promoting older people’s physical and social functioning and in increasing their physical activity in a long-term care environment. Social activity was not measured in any study. Exergames can also be recommended for older people with mild cognitive and physical limitations to promote physical functioning. The effects in terms of psychological functioning are not completely clear, but playing exergames has been able to have a positive effect on the reduction of depressive symptoms. There is a lack of studies that use other kinds of gaming systems, such as mobile games, in interventions for older people. Playing with smart devices and different applications needs further investigation in the future. Implementation of digital gaming activities requires the competence of nursing staff and rehabilitation professionals in digitalization.

## Appendix A

See Table 4.

**Table 4:** Search strategy for every database

Database	Keywords
PubMed	(((“Aged”[Mesh]) OR (aged[Text Word] OR elderly[Text Word] OR senior*[Text Word] OR “older people”[Text Word] OR “older adult*”[Text Word])) AND ((“Video Games”[Mesh]) OR (“digital game*”[Text Word] OR “video game*”[Text Word] OR gaming[Text Word] OR exergaming[Text Word] OR “serious game*”[Text Word]))) AND ((“Residential Facilities”[Mesh]) OR (“nursing home*” [Text Word] OR “rest home*” [Text Word] OR “long-term care” [Text Word] OR institutionalized [Text Word] OR “homes for the aged” [Text Word] OR residental [Text Word] OR residence* [Text Word] OR “Assisted Living” [Text Word] OR facility [Text Word] OR facilities[Text Word])) ((((“Aged”[Mesh]) OR (aged[Text Word] OR elderly[Text Word] OR senior*[Text Word] OR “older people”[Text Word] OR “older adult*”[Text Word])) AND ((“Video Games”[Mesh]) OR (“digital game*”[Text Word] OR “video game*”[Text Word] OR gaming[Text Word] OR exergaming[Text Word] OR “serious game*”[Text Word]))) AND ((((“Activities of Daily Living”[Mesh]) OR “Physical Functional Performance”[Mesh]) OR “Social Interaction”[Mesh]) OR (perform*[Text Word] OR “activities of daily living”[Text Word] OR abilit*[Text Word] OR function*[Text Word] OR cogniti*[Text Word] OR “Independent Living”[Text Word] OR “Social Participation”[Text Word] OR “Social interaction”[Text Word] OR “physical fitness” [Text Word] OR “psychosocial health”[Text Word] OR “social activ*”[Text Word] OR “physical activ*”[Text Word]))) NOT (((“Child”[Mesh]) OR “Adolescent”[Mesh]) OR (child*[Text Word] OR adolescen*[Text Word]))
Web of Science	(“digital game*” OR “video game*” OR gaming OR exergaming OR “serious game*”) and (aged OR elderly OR senior* OR “older people” OR “older adult*”) and (“nursing home*” OR “rest home*” OR “long-term care” OR institutionalized OR “homes for the aged” OR residental OR residence* OR “Assisted Living” OR facility OR facilities) (“digital game*” OR “video game*” OR gaming OR exergaming OR “serious game*”) and (aged OR elderly OR senior* OR “older people” OR “older adult*”) and (perform* OR “activities of daily living” OR abilit* OR function* OR cogniti* OR “Independent Living” OR “Social Participation” OR “Social interaction” OR “physical fitness” OR “psychosocial health” OR “social activ*” OR “physical activ*”) not (child* OR adolescen*)
CINAHL	((MH “Video Games + ”) OR (“digital game*” OR “video game*” OR gaming OR exergaming OR “serious game*”)) AND ( (MH “Aged + ”) OR ( aged OR elderly OR senior* OR “older people” OR “older adult*”)) AND (((MH “Residential Facilities + ”) OR (MH “Assisted Living”)) OR ( “nursing home*” OR “rest home*” OR “long-term care” OR institutionalized OR “homes for the aged” OR residental OR residence* OR “Assisted Living” OR facility OR facilities)) (((MH “Video Games + ”) OR (“digital game*” OR “video game*” OR gaming OR exergaming OR “serious game*”)) AND ((MH “Aged + ”) OR (aged OR elderly OR senior* OR “older people” OR “older adult*”)) AND ((MH “Activities of Daily Living + ”) OR (MH “Physical Performance”) OR (MH “Interpersonal Relations + ”) OR (MH “Physical Activity”) OR perform* OR “activities of daily living” OR abilit* OR function* OR cogniti* OR “Independent Living” OR “Social Participation” OR “Social interaction” OR “physical fitness” OR “psychosocial health” OR “social activ*” OR “physical activ*”)) NOT ((((MH “Adolescence + ”) OR (MH “Child + ”)) OR (child* OR adolescen*))
Scopus	( TITLE-ABS-KEY ( ”digital game*” OR ”video game*” OR gaming OR exergaming OR “serious game*”) AND TITLE-ABS-KEY ( aged OR elderly OR senior* OR “older people” OR “older adult*”) AND TITLE-ABS-KEY ( ”nursing home*” OR “rest home*” OR “long-term care” OR institutionalized OR “homes for the aged” OR residental OR residence* OR “Assisted Living” OR facility OR facilities)) ( TITLE-ABS-KEY ( ”digital game*” OR “video game*” OR gaming OR exergaming OR “serious game*”) AND TITLE-ABS-KEY ( aged OR elderly OR senior* OR “older people” OR “older adult*”) AND TITLE-ABS-KEY ( perform* OR “activities of daily living” OR abilit* OR function* OR cogniti* OR “Independent Living” OR “Social Participation” OR “Social interaction” OR “physical fitness” OR “psychosocial health” OR “social activ*” OR “physical activ*”) AND NOT TITLE-ABS-KEY ( child* OR adolescen*))
